# Trajectories and Adaptive Recalibration of Self‐Efficacy in Brazilian Medical Students: A Longitudinal Study With Implications for Educational Support

**DOI:** 10.1111/tct.70448

**Published:** 2026-06-05

**Authors:** Alex Bertolazzo Quitério, Weslley dos Santos Borges, Ana Maria Rita Pedroso Vilela Torres de Carvalho Engel, João Daniel De Souza Menezes, Emerson Roberto dos Santos, William Donegá Martinez, Matheus Querino da Silva, Stela Regina Pedroso Vilela Torres de Carvalho, Samila Bernardi do Vale Lopes, Maysa Alahmar Bianchin, Vânia Maria Sabadoto Brienze, Janaína Aparecida de Sales Floriano, Júlio César André

**Affiliations:** ^1^ Center for Studies and Development of Health Education – CEDES, São José do Rio Preto Medical School FAMERP São Paulo Brazil; ^2^ Division of Undergraduate Medical Education Brasil University São Paulo Brazil

**Keywords:** longitudinal study, medical education, medical students, professional training, self efficacy

## Abstract

**Introduction:**

Self‐efficacy, the belief in one's capacity to execute actions necessary to achieve specific objectives, is a significant predictor of academic performance and well‐being in medical training. Longitudinal studies examining the dynamic evolution of self‐efficacy throughout undergraduate medical education remain scarce in the Brazilian context. This study prospectively evaluated perceived self‐efficacy among medical students during critical curricular transitions.

**Methods:**

A prospective longitudinal study followed 50 medical students from a Brazilian public university at four time points: course entry (T1–2021), end of first year (T2–2022), midpoint of clinical cycle (T3–2023) and beginning of fifth year/internship (T4–2025). The Self‐Efficacy Scale for Higher Education (SESHE), a validated 34‐item instrument assessing five dimensions, was administered. Statistical analyses included repeated measures ANOVA, Wilcoxon test and effect size calculations (Cohen's *d*).

**Results:**

The study observed a dynamic evolution in perceived self‐efficacy, characterised by a notable shift in total self‐efficacy from 2021 to 2023, followed by partial recovery by 2025. This pattern included significant reductions in several dimensions. Self‐efficacy in proactive actions experienced the most pronounced change (*d* > 0.8), while self‐efficacy in social interaction demonstrated relative stability. Baseline analyses revealed higher self‐efficacy in social interaction among male students (*d* = 0.51).

**Conclusion:**

These findings suggest that the observed changes in self‐efficacy represent an adaptive recalibration, moving toward a more realistic self‐assessment essential for professional development. Therefore, strategies that support this healthy recalibration are crucial. These interventions should aim to facilitate adaptive adjustment, fostering robust and accurate self‐efficacy throughout medical training.

## 1 Introduction

Medical training imposes intense academic, emotional and social demands on students, which continuously challenge their beliefs about their own capacity for success. Understanding how these self‐efficacy beliefs, defined as personal judgements about one's capacity to organise and execute actions necessary to achieve specific goals, evolve during medical undergraduate education has become essential to optimise educational processes and prevent adverse outcomes such as academic burnout and unsatisfactory performance [1, 2, 3]. These beliefs are critical determinants not only of academic achievement and clinical performance but also of resilience and professional identity formation among students [4]. They are not merely reflections of past successes but active shapers of future choices, effort expenditure and perseverance in the face of challenges [1].

Bandura's social cognitive theory [5] postulates that self‐efficacy directly influences motivation, thought patterns, emotional reactions and academic performance [5]. Students with high self‐efficacy tend to persist in the face of obstacles, employ self‐regulated learning strategies and demonstrate greater academic engagement [6]. Conversely, low levels of self‐efficacy are associated with procrastination, anxiety and lower performance [7]. In medical education, evidence consolidates self‐efficacy as a predictor not only of academic success but also of psychological well‐being and preparedness for clinical practice [8, 9]. Critically, self‐efficacy is a multidimensional construct, varying in strength, generality and level [1]. It is not a global trait but domain‐specific, meaning individuals may possess high self‐efficacy in one area (e.g., clinical skills) and lower self‐efficacy in another (e.g., stress management). In the context of medical training, specific facets of self‐efficacy are particularly salient, encompassing clinical judgement and decision‐making, communication skills, academic self‐regulation, proactive professional development and coping with stress [4, 9, 10]. By disaggregating self‐efficacy into these distinct yet interrelated domains, this study aims to provide a nuanced understanding of how specific self‐beliefs evolve and how they are operationalised in our current investigation. Self‐efficacy in clinical judgement and decision‐making is paramount for medical students' competence and confidence in patient care, with research consistently highlighting its importance for effective patient interactions [11]. Effective communication is a cornerstone of medical practice, and self‐efficacy in this domain correlates directly with better patient outcomes and professional relationships, as demonstrated in studies showing its link to core nursing competencies [4, 9, 12]. The ability to manage one's learning process is vital, with self‐efficacy in academic self‐regulation predicting better learning outcomes and adaptability, especially in technology‐enhanced learning environments [6]. Engaging in continuous professional growth is essential for medical practitioners. Self‐efficacy in proactive professional development has been linked to increased participation in scholarly activities and career advancement, with higher self‐efficacy among medical students correlating with greater involvement in research [9, 13, 14]. Managing stress effectively is critical in the demanding field of medicine. Self‐efficacy in stress coping strategies has been associated with lower burnout rates and better mental health among medical students, equipping them better to handle training stresses [3, 15, 16].

*Self‐efficacy in clinical judgement and decision‐making is paramount for medical students' competence*.

Recent systematic reviews document the global relevance of self‐efficacy in medical education, associating it with the development of multidimensional competencies, effective learning strategies and positive emotional states [4, 10]. However, longitudinal studies, capable of capturing individual trajectories and identifying critical moments of vulnerability during curricular transitions, remain scarce [17]. Such designs are crucial for understanding the dynamic nature of self‐efficacy development throughout the rigorous and transformative journey of medical education [18]. While cross‐sectional studies offer valuable snapshots, they cannot adequately capture the developmental trajectories or the interplay of factors influencing self‐efficacy over time. Previous longitudinal research in medical education, primarily conducted in North America and Europe, has yielded mixed findings regarding self‐efficacy trends, with some studies reporting stability or gradual increases, while others suggest declines, particularly during transitions to clinical years [4, 9, 10]. These variations often depend on the specific domains of self‐efficacy measured, the curricular structure and the cultural context of the educational institution. Building upon these findings, it is reasonable to hypothesise that while certain aspects of self‐efficacy, such as academic self‐regulation, might initially decline as students confront increasing complexity, others, like clinical judgement and communication skills, might gradually increase with direct experience and skill acquisition. This study aims to explore these differential trajectories. This methodological gap is particularly relevant considering that transitions between cycles (basic, clinical and internship) represent periods of academic and identity reorganisation that can intensify psychosocial challenges and substantially modify self‐efficacy beliefs [19].

Beyond the Brazilian context, several longitudinal studies have explored self‐efficacy trajectories among medical students and in related disciplines. For instance, Padiko et al. [20] qualitatively investigated the impact of early, longitudinal community‐based education on medical student self‐efficacy, highlighting the benefits of patient interaction from the first to seventh semester. Similarly, Lurie et al. [21] examined changes in self‐perceived abilities among medical students after their first year of clinical training, noting gender differences in self‐assessment. In nursing education, Bulfone et al. [22] conducted a longitudinal analysis of academic self‐efficacy changes over time, identifying predictive variables influencing these changes. Additionally, Whitcomb et al. [23] studied self‐efficacy among undergraduate women in engineering, revealing a mismatch between self‐efficacy and performance, with women exhibiting lower self‐efficacy despite higher grades. These studies underscore the importance of longitudinal designs in capturing the dynamic nature of self‐efficacy across various educational contexts and among diverse student populations, offering insights into how efficacy beliefs related to academic performance, professional skills and personal development evolve [20, 21, 22, 23].

In the Brazilian context, marked by institutional heterogeneity and recent transformations in the profile of medical students [24], longitudinal investigations on self‐efficacy are practically nonexistent. This gap is significant given the unique cultural, socio‐economic and healthcare system contexts that may influence students' experiences and self‐efficacy development [24]. Understanding these trajectories within the Brazilian medical education landscape is crucial for tailoring culturally appropriate educational strategies and requires considering local specificities. Evidence‐based interventions to strengthen self‐efficacy, including psychosocial support programs, metacognitive strategies [25] and self‐regulation development, require a deep understanding of the temporal trajectories of this construct [7, 26, 27].

Moreover, while self‐efficacy studies often control for demographic variables, the rationale for their inclusion is frequently implicit. This study explicitly investigates the influence of sociodemographic factors such as biological sex, gender identity, income and type of prior schooling (public vs. private education). These variables are critical within the Brazilian context, where historical and systemic inequalities significantly impact access to education, educational experiences and professional trajectories [28]. For instance, students from public versus private school backgrounds may enter medical school with differential preparatory advantages or disadvantages, potentially affecting their initial self‐efficacy levels and subsequent development. Similarly, gender identity and income can influence students' experiences of belonging, perceived support and exposure to different learning opportunities, all of which are salient sources of self‐efficacy [1, 29]. Therefore, by foregrounding these sociodemographic dynamics, we aim to provide a more comprehensive and contextualised understanding of self‐efficacy development.

Central to reframing our interpretation of declining self‐efficacy trajectories is the integration of calibration theory with the Dreyfus model of skill acquisition [30]. This is not merely descriptive language added post hoc to our findings; rather, it functions as an active theoretical lens that fundamentally reinterprets what our longitudinal data represent. Under this framework, initial high self‐efficacy does not signal preparedness but reflects unconscious incompetence—a well‐documented cognitive state preceding genuine competence development. The observed decline in our study becomes theoretically intelligible not as educational failure but as evidence of adaptive recalibration: Students confront authentic clinical complexity, receive reality‐grounded feedback and reorganise their self‐beliefs toward conscious incompetence and, subsequently, toward conscious competence [30, 31]. This theoretical reorientation is not semantic adjustment; it restructures how we analyse and interpret the very patterns in our data (Tables 2, 3, 4 and Figure 4) and what educational implications logically follow.

*Central to reframing our interpretation of declining self‐efficacy trajectories is the integration of calibration theory*.

Central to our investigation is the understanding of self‐efficacy changes not merely as gains or losses but as potential developmental transitions or processes of recalibration. Contemporary scholarship in Health Professions Education (HPE) increasingly recognises the importance of concepts like calibration, professional identity formation, workplace learning and entrustment ([4] a). From this perspective, an initial high level of self‐efficacy, often seen in novice learners, may reflect ‘unconscious incompetence’ or a lack of familiarity with the true demands of the profession. As students progress, confront increasing complexity, receive authentic feedback and move from ‘conscious incompetence’ toward ‘conscious competence’, their self‐efficacy beliefs may undergo a necessary recalibration, potentially appearing as a temporary decline. This recalibration is not necessarily problematic; instead, it can be a healthy and adaptive response to the growing cognitive and emotional demands of training, leading to a more accurate and robust self‐assessment necessary for professional development and ultimate entrustment [30].

**FIGURE 1:**
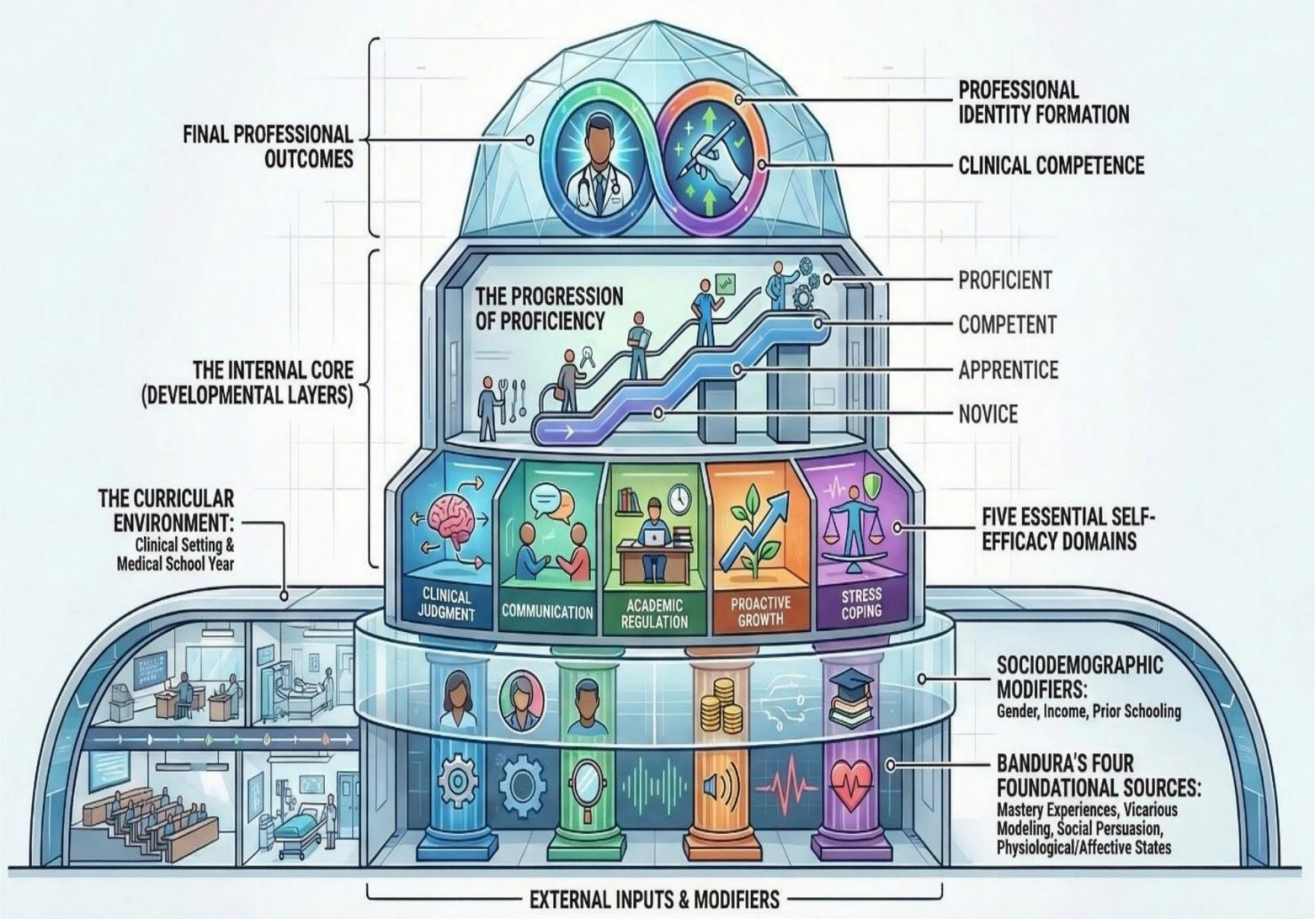
Theoretical framework illustrating the dynamic interplay between self‐efficacy beliefs and various influencing factors throughout medical education. *Source:* Author.

The theoretical framework guiding this study integrates Bandura's Social Cognitive Theory [1] with contemporary constructs from HPE, emphasising the dynamic, multidimensional and contextual nature of self‐efficacy development. This framework posits that students' self‐efficacy beliefs are shaped by multiple sources of influence (mastery experiences, vicarious experiences, social persuasion, physiological and affective states) and are continuously recalibrated as they navigate the curriculum and clinical environments. We propose a model where self‐efficacy acts as a mediator in the development of professional identity and clinical competence, while also being influenced by sociodemographic factors and the specific demands of each phase of medical training. Figure 1 illustrates this integrated theoretical framework [1].

This study addresses a critical gap in the literature by longitudinally exploring self‐efficacy trajectories across four strategic moments of undergraduate medical education in Brazil. Specifically, it aimed to (1) characterise baseline self‐efficacy levels across five specific domains (clinical judgement and decision‐making, communication skills, academic self‐regulation, proactive professional development and coping with stress) and their relationship with sociodemographic variables; (2) analyse longitudinal changes and patterns in these five self‐efficacy domains over time, considering the potential for recalibration and developmental transitions; and (3) identify heterogeneous patterns of individual trajectories and interpret the observed changes within the broader context of professional identity formation and the dynamic demands of medical training. The results can inform the development of institutional student support strategies that acknowledge the developmental nature of self‐efficacy and are aligned with global demands for excellence in medical education.

## 2 Methods

### 2.1 Study Design and Population

A descriptive, longitudinal, prospective and quantitative study [32] was conducted to evaluate perceived self‐efficacy among medical students at the School of Medicine of São José do Rio Preto (FAMERP). The target population included all 80 students enrolled in 2021. The final sample consisted of 50 participants who completed all four longitudinal assessments, resulting in a retention rate of 62.5%. Inclusion criteria were the following: regular enrollment in the medical program at FAMERP, admission in 2021 and informed consent. Participants were excluded for withdrawal from the course or failure to complete all assessments.

### 2.2 Data Collection Periods

Data were collected at the following four critical time points in the medical curriculum:

- T1 (2021): Course initiation (basic science phase)
- T2 (2022): End of second year (completion of basic science phase)
- T3 (2023): End of third year (midpoint of clinical phase)
- T4 (2025): Beginning of fifth year (entry into clinical internship)

This longitudinal approach was based on the understanding that curricular transitions represent critical periods for self‐efficacy beliefs [1, 18].

Students were recruited through institutional email invitations, which were developed and sent by the research team. The surveys were administered online using standardised questionnaires. The average completion time for each assessment was between 15 and 20 min. This duration was necessary due to the instrument's 34 items and the inherent complexity of the questions, which required thoughtful reflection on various aspects of self‐efficacy. In addition to the self‐efficacy measure, the questionnaire included additional items to collect sociodemographic variables, such as biological sex, gender identity, age, sexual orientation, marital status, number of children, income, religion, type of school attended during elementary and secondary education and paid employment status.

### 2.3 Self‐Efficacy Measurement

Self‐efficacy was measured using the SESHE [33], an instrument validated for the Brazilian context that demonstrates adequate construct validity and reliability coefficients (Cronbach's alpha) exceeding 0.70. The scale comprises 34 items distributed across five dimensions:

- Academic Self‐Efficacy (nine items): Evaluates students' confidence in their academic abilities, such as comprehending complex concepts and applying knowledge in exams.
- Self‐Efficacy in Formation Regulation (seven items): Measures students' capacity for self‐regulation regarding their learning process, including planning and organising studies.
- Self‐Efficacy in Social Interaction (seven items): Reflects students' confidence in their communication and interaction skills with peers and professors.
- Self‐Efficacy in Proactive Actions (six items): Assesses students' initiative in seeking opportunities for learning and professional development
- Self‐Efficacy in Academic Management (five items): Measures students' ability to manage academic demands, such as meeting deadlines and handling multiple tasks.

Items are responded to on a 10‐point Likert scale (1 = *completely incapable* to 10 = *completely capable*). The total score is calculated as the sum of all dimension scores. Examples of items include: ‘I am able to understand complex concepts presented in classes’, ‘I can organise my study time efficiently’ and ‘I feel confident interacting with professors during classes’. For the current sample, the internal consistency of the scale was assessed, yielding Cronbach's alpha coefficients greater than 0.75 for all dimensions, indicating good reliability.

### 2.4 Ethical Procedures and Data Collection

The project received approval from the FAMERP Research Ethics Committee (Opinion n° 41220020.8.0000.5415), in accordance with Resolution 466/2012 of the National Health Council and the Declaration of Helsinki (World Medical Association., 2013). All participants provided written informed consent. The confidentiality of data was ensured through the anonymisation of responses and secure storage of collected information.

### 2.5 Data Analysis

#### 2.5.1 Descriptive Statistics

Sociodemographic variables were presented as absolute and relative frequencies. For self‐efficacy scores, measures of central tendency (means and medians) and dispersion (standard deviations and interquartile ranges) were calculated. Scores were categorised as weak (percentile ≤ 25), moderate (percentile 25–75) and strong (percentile ≥ 75) [33].

#### 2.5.2 Normality Tests

Data normality was assessed using the Shapiro–Wilk test, which indicated non‐normality (*p* < 0.001). Despite this, repeated measures ANOVA was chosen for longitudinal analyses due to its established robustness to violations of normality, particularly in samples of moderate to large size, as supported by statistical literature [34]. While alternative approaches like longitudinal mixed‐effects models could offer additional insights into intrasubject clustering and simultaneous examination of time and demographic predictors, the decision to maintain repeated measures ANOVA was based on the adequacy of the method for the specific research questions and its established robustness under the observed data characteristics.

#### 2.5.3 Baseline Comparative Analyses

The Wilcoxon rank‐sum test (Mann–Whitney *U*) was employed to compare self‐efficacy scores at T1 between sociodemographic groups [35]. Effect size was estimated using the point‐biserial correlation coefficient (r‐biserial), interpreted as small (0.10–0.29), medium (0.30–0.49) and large (≥ 0.50) [36].

#### 2.5.4 Longitudinal Analyses

Temporal changes in self‐efficacy were evaluated using repeated measures ANOVA, with the four assessment time points as the within‐subjects factor [37]. Prior to analysis, the assumption of sphericity was assessed using Mauchly's test. When violations of sphericity occurred, Greenhouse–Geisser or Huynh‐Feldt corrections were applied to adjust the degrees of freedom [37]. Post hoc tests with Bonferroni correction identified pairwise differences between time points, controlling for Type I error. Effect size was calculated using Cohen's *d* for paired samples, interpreted as small (0.20–0.49), medium (0.50–0.79) and large (≥ 0.80) [36]. The inclusion of sociodemographic predictors in the baseline comparative analyses (and their exploration in the introduction) was motivated by their critical role in shaping educational experiences and self‐efficacy development within the Brazilian context, addressing historical and systemic inequalities as highlighted by prior research [1, 28].

#### 2.5.5 Individual Trajectory Analysis

A descriptive analysis of individual trajectories was conducted to identify heterogeneous patterns of change over RIStime.

#### 2.5.6 Significance Level

A significance level of *α* = 0.05 was adopted for all statistical tests, performed using SPSS version 25.0 software (IBM Corp., 2017).

### 2.6 Attrition Rate Analysis

The study's retention rate was 62.5%, with 50 out of 80 initial participants completing all four assessments. To investigate potential attrition bias, comparisons of baseline sociodemographic characteristics were performed between participants who completed the study and those who were lost to follow‐up. No statistically significant differences were found between these groups, suggesting that participant loss did not introduce significant bias into the results.

## 3 Results

This section presents the findings of the longitudinal analysis of perceived self‐efficacy among medical students. The progression includes sociodemographic characterisation of the sample, descriptive evolution of self‐efficacy scores, baseline comparative analyses and finally, longitudinal inferential analyses with individual trajectories.

### 3.1 Sociodemographic Characterisation of the Cohort

The longitudinal cohort of 50 medical students admitted in 2021 at FAMERP revealed a predominantly young profile (76% aged between 0 and 20 years), predominantly male (56% biological sex and 54% identifying as men). The vast majority of participants were heterosexual (82%), single (98%) and childless (98%). Regarding socioeconomic status, 82% reported personal or family income in the range of R$ 1001.00 to R$ 3000.00. Prior education was predominantly in private institutions (74% for elementary school and 76% for secondary school). It is noteworthy that 98% of students were not engaged in paid employment at course entry. Concerning religious affiliation, 54% reported having no religion, followed by 36% Catholic, 6% Evangelical and 2% Afro‐Brazilian or Eastern religions. The sociodemographic data characterising the sample are summarised in Table 1.

**TABLE 1:** Sample characterisation—sociodemographic variables (Brazil, 2025).

Variable	*N* = 50^a^
Gender biological	
Female	22 (44)
Male	28 (56)
Gender identity	
Female	23 (46)
Male	27 (54)
Sexual orientation	
Heterosexual	41 (82)
Bisexual	3 (6)
Homosexual	6 (12)
Age group	
0–20 years	38 (76)
21–30 years	11 (22)
31–40 years	1 (2)
Marital status	
Single	49 (98)
Married/stable union	1 (2)
Number of children	
None	49 (98)
4	1 (2)
Income range (Brazilian Reais – R$)	
≤ 1.000.00	9 (18)
1.001.00 a 3.000.00	41 (82)
Religion	
No religion	27 (54)
Afro‐Brazilian religion	1 (2)
Catholic	18 (36)
Protestant	3 (6)
Eastern religions	1 (2)
Primary education	
All or most of it in public school	13 (26)
All or most of it in private school	37 (74)
School type – primary education	
Regular course (8 years)	50 (100)
High school	
All or most of it in public school	12 (24)
All or most of it in private school	38 (76)
School type – high school	
Regular (3 years)	48 (96)
Technical school	2 (4)
Weekly working hours	
None	49 (98)
4 h	1 (2)

^a^ *n* (%).

### 3.2 Evolution of Perceived Self‐Efficacy Throughout Medical Training

Descriptive analysis of the SESHE revealed a generalised decrease in mean scores across all dimensions and in the total score from 2021 to 2023, followed by slight recovery in 2025, without, however, restoring initial levels. Non‐normality of data was confirmed (*p* < 0.001). Table 2 categorises scores into ‘weak’, ‘moderate’ and ‘strong’ across time points.

**TABLE 2:** Categories of perceived self‐efficacy in medical students throughout the basic, clinical and internship cycles (Brazil, 2025).

Variable	2021 *N* = 50^a^	2022 *N* = 50^a^	2023 *N* = 50^a^	2025 *N* = 50^a^
Academic self‐efficacy				
Weak	0 (0)	4 (8)	1 (2)	0 (0)
Moderate	7 (14)	16 (32)	26 (52)	19 (38)
Strong	43 (86)	30 (60)	23 (46)	31 (62)
Self‐efficacy in learning regulation				
Weak	0 (0)	6 (12)	7 (14)	2 (4)
Moderate	9 (18)	20 (40)	23 (46)	23 (46)
Strong	41 (82)	24 (48)	20 (40)	25 (50)
Self‐efficacy in social interaction				
Weak	1 (2)	5 (10)	3 (6)	1 (2)
Moderate	7 (14)	19 (38)	23 (46)	14 (28)
Strong	42 (84)	26 (52)	24 (48)	35 (70)
Self‐efficacy in proactive actions				
Weak	0 (0)	9 (18)	14 (28)	17 (34)
Moderate	8 (16)	21 (42)	22 (44)	20 (40)
Strong	42 (84)	20 (40)	14 (28)	13 (26)
Self‐efficacy in academic management				
Weak	0 (0)	2 (4)	2 (4)	2 (4)
Moderate	1 (2)	10 (20)	10 (20)	8 (16)
Strong	49 (98)	38 (76)	38 (76)	40 (80)
Total score				
Weak	0 (0)	2 (4)	3 (6)	0 (0)
Moderate	3 (6)	21 (42)	26 (52)	25 (50)
Strong	47 (94)	27 (54)	21 (42)	25 (50)

^a^ *n* (%).

**FIGURE 2:**
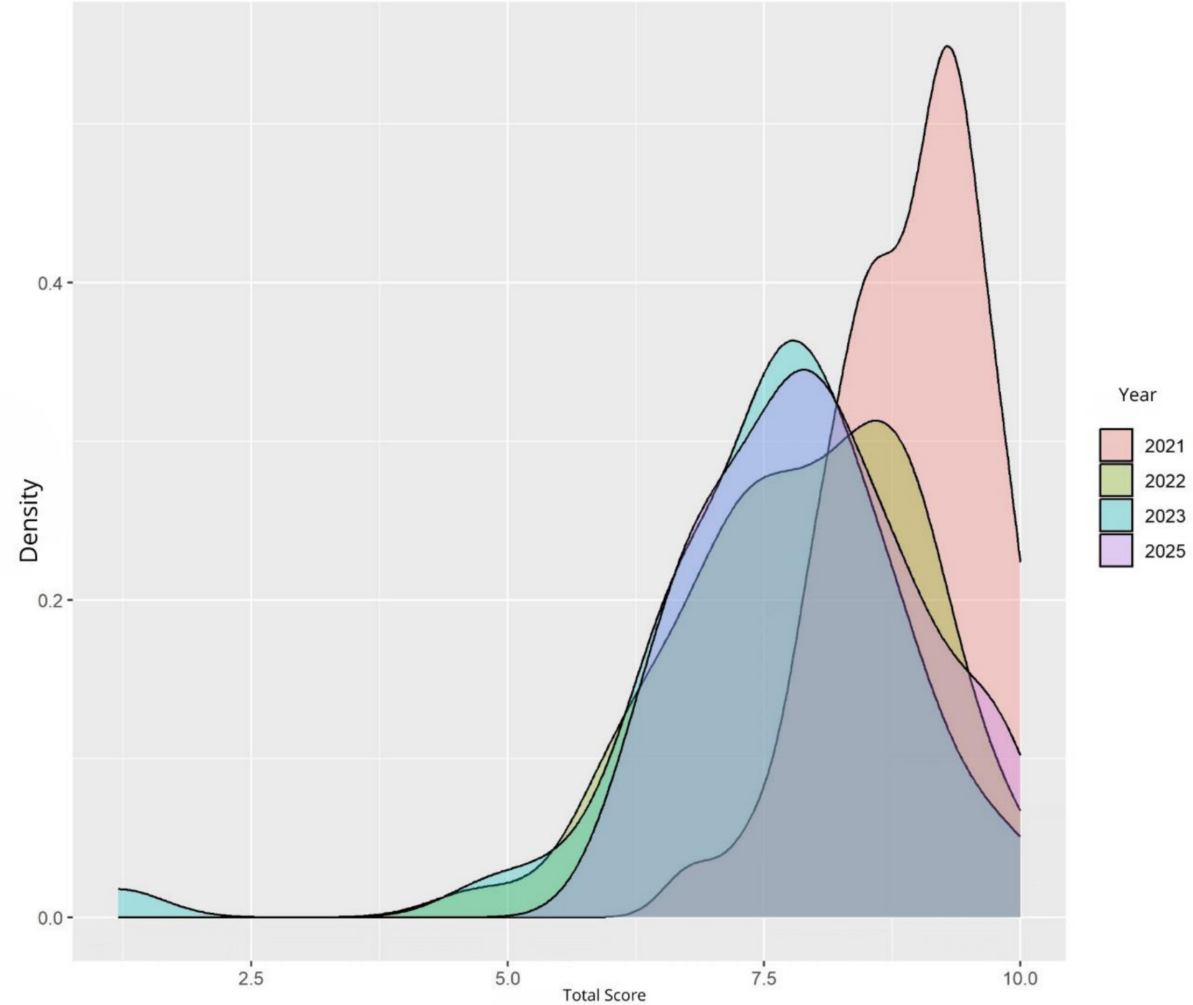
Distribution of the total self‐efficacy score among medical students at different stages of their undergraduate studies (Brazil, 2025). *Source:* Author.

Table 2 demonstrates that the proportion of students with ‘strong’ self‐efficacy in the total score declined from 94% (2021) to 50% (2025), with the ‘moderate’ category increasing from 6% to 50%. The self‐efficacy in proactive actions dimension was the most affected, with the ‘strong’ category declining from 84% to 26% and the ‘weak’ category increasing from 0% to 34% during the same period. Figure 2 illustrates the decline in the total score and reveals a trajectory of decline in total self‐efficacy score from 2021 to 2023, followed by partial recovery in 2025, which does not reach initial levels.

### 3.3 Comparative Analysis by Sociodemographic Variables

The Wilcoxon (Mann–Whitney *U*) test indicated that only Self‐Efficacy in Social Interaction showed a statistically significant difference between biological sexes (*p* = 0.032), with higher median scores for males. The r‐biserial effect size of 0.305 suggested a medium magnitude [36]. No other sociodemographic variables (gender identity, income, school type) showed significant differences (*p* > 0.05), with small effect sizes. Table 3 summarises these findings.

**TABLE 3:** Comparison of median self‐efficacy scores by biological sex at baseline (Brazil, 2025).

Variable	Female *N* = 22^a^	Male *N* = 28^a^	*p* ^b^	ES [IC 95%]
Academic self‐efficacy	8.83 (8.22, 9.33)	8.89 (8.56, 9.78)	0.252	0.163 [−0.07; 0.37]
Self‐efficacy in learning regulation	8.86 (8.00, 9.43)	8.79 (8.50, 9.43)	0.961	0.008 [−0.25; 0.07]
Self‐efficacy in social interaction	8.43 (8.14, 8.86)	8.79 (8.43, 9.43)	0.032	0.305 [0.05; 0.56]
Self‐efficacy in proactive actions	8.79 (8.00, 9.29)	9.14 (8.21, 9.79)	0.161	0.2 [−0.03; 0.42]
Self‐efficacy in academic management	9.50 (9.00, 10.00)	9.50 (8.75, 10.00)	0.936	0.013 [−0.25; 0.08]
Total score	8.93 (8.19, 9.38)	9.19 (8.53, 9.51)	0.353	0.133 [−0.1; 0.32]

Abbreviation: ES, effect size (r‐biserial).

^a^ Median (Q1, Q3).

^b^ Wilcoxon rank‐sum test.

### 3.4 Longitudinal Inferential Analysis: Impact of Time on Self‐Efficacy

Repeated measures analysis of variance (ANOVA) was conducted to evaluate the temporal changes in the five self‐efficacy dimensions (academic self‐efficacy, self‐efficacy in formation regulation, self‐efficacy in social interaction, self‐efficacy in proactive actions and self‐efficacy in academic management) and the total self‐efficacy score. All dimensions and the total score demonstrated statistically significant changes over time (*p* < 0.001). Detailed *F*‐statistics, *p*‐values for the main effect of time and Cohen's *d* values for Bonferroni‐corrected post hoc pairwise comparisons are presented in Table 4. These analyses revealed distinct patterns of change across the assessment periods:

- Academic Self‐Efficacy: A statistically significant effect of time was observed. Post hoc comparisons indicated large magnitude reductions from 2021 to 2022 (*d* = 0.822), 2021 to 2023 (*d* = 1.075) and 2021 to 2025 (*d* = 0.799). No significant differences were found between subsequent years (e.g., 2022, 2023 and 2025), suggesting that the primary decline occurred early in the curriculum.
- Self‐Efficacy in Formation Regulation: A statistically significant effect of time was observed. Large magnitude reductions occurred between 2021 and 2022 (*d* = 0.863) and 2021 and 2023 (*d* = 1.195). A moderate magnitude reduction (*d* = 0.588) was observed between 2021 and 2025. No significant differences were found among 2022, 2023, and 2025.
- Self‐Efficacy in Social Interaction: A statistically significant effect of time was observed. Moderate to large magnitude differences occurred between 2021 and 2022 (*d* = 0.775) and 2021 and 2023 (*d* = 0.798). No significant differences were found when comparing 2021 to 2025 or among 2022, 2023 and 2025, suggesting greater stability in this dimension after an initial decline.
- Self‐Efficacy in Proactive Actions: This dimension exhibited the most robust and statistically significant temporal effect. Significant and large magnitude reductions were consistent across all comparisons with 2021: 2021–2022 (*d* = 1.100), 2021–2023 (*d* = 1.363) and 2021–2025 (*d* = 1.359). No significant differences were found among 2022, 2023 and 2025.
- Self‐Efficacy in Academic Management: A statistically significant effect of time was observed. Significant reductions were found between 2021 and 2022 (*d* = 0.742, moderate), 2021 and 2023 (*d* = 0.913, large) and 2021 and 2025 (*d* = 0.751, moderate). No significant differences were found among 2022, 2023 and 2025.
- Total Self‐Efficacy Score: A statistically significant effect of time was observed. Post hoc analyses revealed significant and large magnitude reductions in all comparisons with the year of admission: 2021–2022 (*d* = 1.011), 2021–2023 (*d* = 1.268) and 2021–2025 (*d* = 0.934). No significant differences were found among 2022, 2023 and 2025.

In summary, longitudinal analyses demonstrate robust and large magnitude reduction in perceived self‐efficacy among medical students, primarily in the transition from the year of admission to subsequent years. Self‐efficacy levels do not return to initial levels, with self‐efficacy in proactive actions being the most consistently and severely affected dimension. The large effect sizes [36] underscore the practical relevance of these changes. Overall, longitudinal analyses consistently revealed statistically significant declines in perceived self‐efficacy among medical students across all assessed dimensions, with the most substantial reductions observed in the transition from the year of admission to subsequent years. Notably, self‐efficacy levels did not recover to initial baseline levels by the final assessment point in 2025. The dimension of self‐efficacy in proactive actions experienced the most consistent and pronounced magnitude of decline across the study period.

**TABLE 4:** Longitudinal changes in self‐efficacy dimensions and total score (repeated measures ANOVA main effects and Bonferroni‐corrected post hoc pairwise comparisons) (Brazil, 2025).

Self‐efficacy dimension	Main effect of time (*F*(df1, df2), *p*)	2021 vs. 2022 (*d*)	2021 vs. 2023 (*d*)	2021 vs. 2025 (*d*)
Academic self‐efficacy	*F*(3, 147) = 11.295, *p* < 0.001	0.822	1.075	0.799
Self‐efficacy in formation regulation	*F*(3, 147) = 13.512, *p* < 0.001	0.863	1.195	0.588
Self‐efficacy in social interaction	*F*(3, 147) = 7.908, *p* < 0.001	0.775	0.798	NS
Self‐efficacy in proactive actions	*F*(3, 147) = 23.939, *p* < 0.001	1.100	1.363	1.359
Self‐efficacy in academic management	*F*(3, 147) = 8.888, *p* < 0.001	0.742	0.913	0.751
Total self‐efficacy score	*F*(3, 147) = 16.696, *p* < 0.001	1.011	1.268	0.934

*Note:* NS indicates no statistically significant difference in the pairwise comparison.

*Longitudinal analyses demonstrate robust and large magnitude reduction in perceived self‐efficacy among medical students*.

### 3.5 Analysis of Individual Trajectories of Self‐Efficacy

**FIGURE 3:**
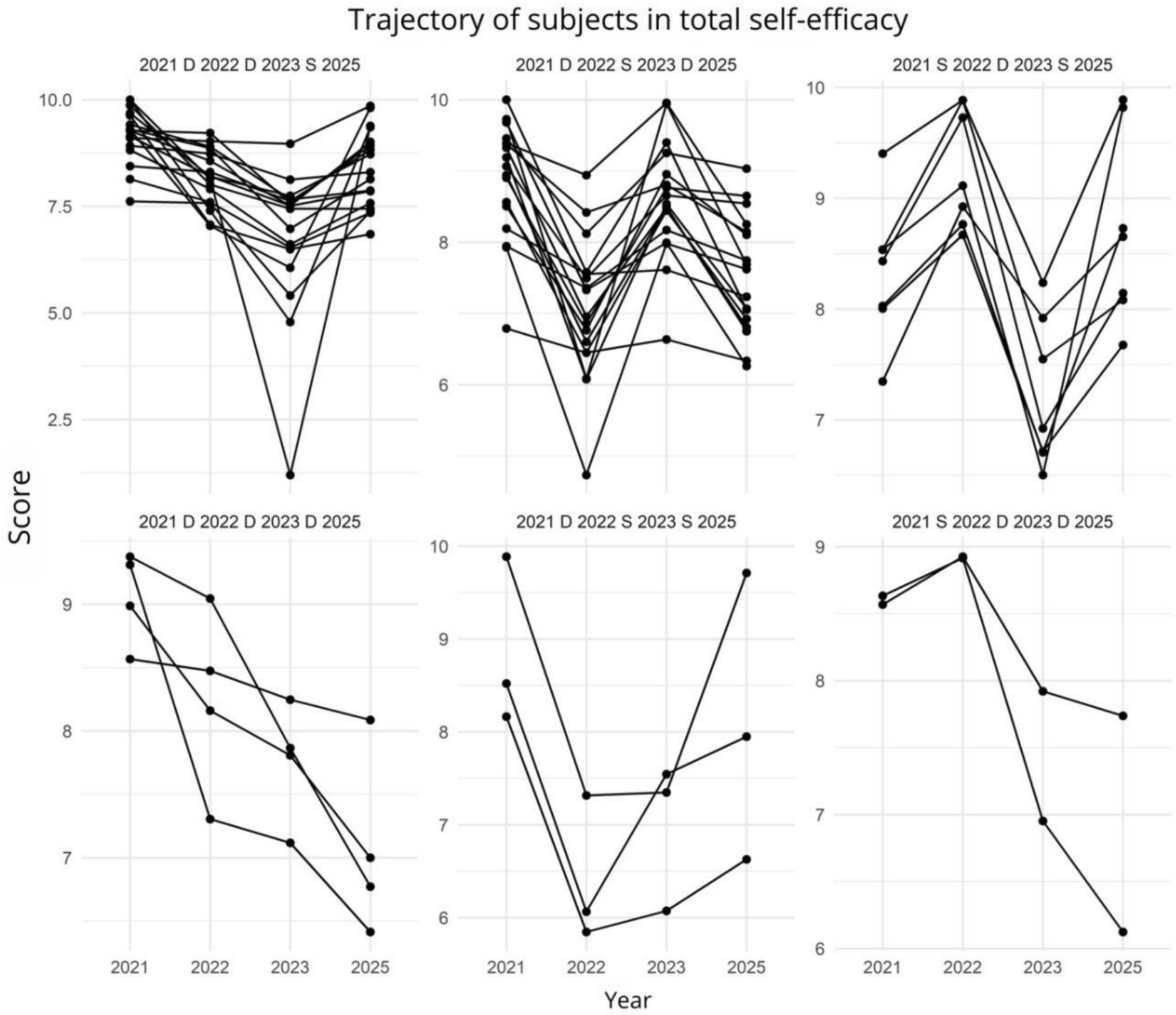
Individual trajectories of total self‐efficacy scores in medical students throughout their undergraduate studies, indicating patterns of decrease (D), increase (S) or stability (I) in scores between 2021, 2022, 2023 and 2025 (Brazil, 2025). *Source:* Author.

Descriptive analysis of individual trajectories revealed notable heterogeneity in patterns of self‐efficacy change, contrasting with group mean trends. Figure 3, a composite figure, illustrates the complexity of these journeys, where D (decreased), S (increased) and E (equal) indicate score variation between consecutive years for each dimension and the total score.

*Individual trajectories revealed notable heterogeneity in patterns of self‐efficacy change, contrasting with group mean trends*.

Figure 3 highlights that the individual experience of self‐efficacy is not uniform; some students maintain or strengthen their beliefs, while others face sharp and persistent declines. This variability emphasises the singular influence of intrinsic and extrinsic factors on the perception of individual competence throughout demanding medical training. This variability highlights that individual experiences of self‐efficacy change are diverse, with some students maintaining or strengthening their beliefs while others face sustained declines, as depicted in Figure 3.

## 4 Discussion

This prospective longitudinal study revealed a significant and sustained shift in perceived self‐efficacy among medical students over 4 years of training. While initial levels were high, a consistent decline was observed, particularly between course entry and the midpoint of the clinical cycle, followed by a partial recovery by the beginning of the internship. This trajectory sheds light on a dynamic phenomenon that requires careful interpretation within the context of demanding professional education. Understanding the evolution of self‐efficacy beliefs is fundamental, as it directly influences persistence, engagement and academic performance, and is a predictive construct of success in medical training [16]. Recent studies further demonstrate that social cognitive factors, including self‐efficacy, explain a substantial proportion of variance in academic satisfaction among medical students, emphasising their relevance for well‐being and professional development [13]. The magnitude of the effect observed, predominantly large (*d* > 0.8) across all SESHE dimensions, indicates that these changes possess significant practical relevance for medical education [33, 36].

These large effect sizes (*d* = 1.27 for total self‐efficacy by 2023) are theoretically coherent with calibration and Dreyfus models. Under calibration theory, the sharp decline between 2021 and 2023 (Table 4) reflects the transition from unconscious incompetence (inflated initial beliefs, 94% ‘strong’ in 2021) to conscious incompetence (halved to 42% by 2025, with 50% in ‘moderate’ range). This is not measurement error or curricular deficiency; it is the predicted temporal signature of cognitive reorganisation during skill acquisition. The dimension most severely affected—self‐efficacy in proactive actions (*d* = 1.36)—aligns perfectly with Dreyfus predictions: Proactivity assumes confidence in one's capacity to initiate action, which necessarily decreases when students confront the vastness and complexity of clinical domains previously unknown. The relative stability of self‐efficacy in social interaction (*d* = 0.80, no recovery to baseline by 2025) conversely reflects the protective role of peer and relational experiences, which remain accessible and reinforcing throughout training. In sum, our data do not merely show decline; they demonstrate the characteristic temporal pattern predicted by recalibration theory, validating the theoretical reinterpretation of these longitudinal trajectories.

The observed pattern of decline in self‐efficacy, followed by a partial recovery, contributes to filling gaps in the literature, particularly given the scarcity of longitudinal studies in the Brazilian context. While cross‐sectional investigations offer isolated snapshots, they cannot adequately capture the developmental trajectories or the interplay of factors influencing self‐efficacy over time. This study, by employing a longitudinal design, provides a more nuanced understanding, demonstrating that the pattern of change is not uniform among individuals, suggesting the existence of both protective and risk factors that modulate responses to academic demands. Specifically, the initial high levels of self‐efficacy, particularly at course entry, may reflect what is termed ‘unconscious incompetence’, a lack of familiarity with the true demands and complexities of the medical profession [30]. As students progress and confront increasing complexity, they receive authentic feedback and move from this initial state toward ‘conscious incompetence’, necessitating a recalibration of their self‐efficacy beliefs [31]. From this perspective, the initial decline observed in our study is not necessarily problematic; instead, it can be a healthy and adaptive response to the growing cognitive and emotional demands of training, leading to a more accurate and robust self‐assessment necessary for professional development and ultimate entrustment [38]. This interpretation aligns with contemporary scholarship in HPE that emphasises concepts like calibration, professional identity formation, workplace learning and developmental transitions [39].

The self‐efficacy in proactive actions dimension exhibited the most pronounced decline. While proactivity is indeed fundamental for contemporary medical practice, this notable reduction could be interpreted as a significant recalibration in response to the growing awareness of the vast and complex landscape of medical education and professional development [1]. From the perspective of social cognitive Theory, self‐efficacy is constructed primarily through experiences of success [1, 5]. As medical students' efficacy beliefs are acquired based on cognitive processing and interpretation of diverse experiences, enactive (direct success), vicarious (observation of others), verbal persuasions and physiological reactions to stressful situations [9], curricular overload and exposure to complex clinical situations may have led to a more realistic perception that new demands are significantly more challenging than initially expected or that previous strategies and capacities are no longer fully adequate [40, 41].

*The self‐efficacy in proactive actions dimension exhibited the most pronounced decline*.

It is important to note that self‐efficacy beliefs, once developed, significantly influence motivation, patterns of thinking and emotional reactions [1]. Studies demonstrate structural relationships between self‐efficacy, academic motivation, self‐regulated learning strategies and academic performance among medical students ([6] b). Further research by Kubrusly et al. [17] in hybrid curricula suggests that self‐efficacy is influenced by a multitude of associated factors, indicating the dynamic and context‐dependent nature of this construct [17].

The partial recovery observed during internship, while not reaching initial inflated levels, may be interpreted as a process of adaptation and consolidation of new mastery experiences. This is a crucial period where students apply theoretical knowledge in real‐world scenarios, receive more immediate and direct feedback and begin to perceive the tangible value of their contribution. Reference [19] characterised medical internship as a significant and meaningful transition, which provides a framework for understanding this observed recovery. This period allows for the development of new mastery experiences, a key source of self‐efficacy according to Bandura [1], as students successfully navigate increasingly complex clinical challenges [1].

The maintenance of relatively stable levels in self‐efficacy in social interaction throughout the course contrasts with the decline in other dimensions, suggesting that interpersonal relationships established during undergraduate training may function as a protective factor. This stability in social self‐efficacy points to the continuous role of social persuasion and vicarious experiences—other significant sources of self‐efficacy [1]—derived from interactions with peers and professors. The literature supports that peer support can positively impact empathy and self‐efficacy among students [42]. This finding reinforces the importance of educational environments that foster peer social support, allowing students to observe and learn from each other's successful interactions and receive encouraging feedback.

**FIGURE 4:**
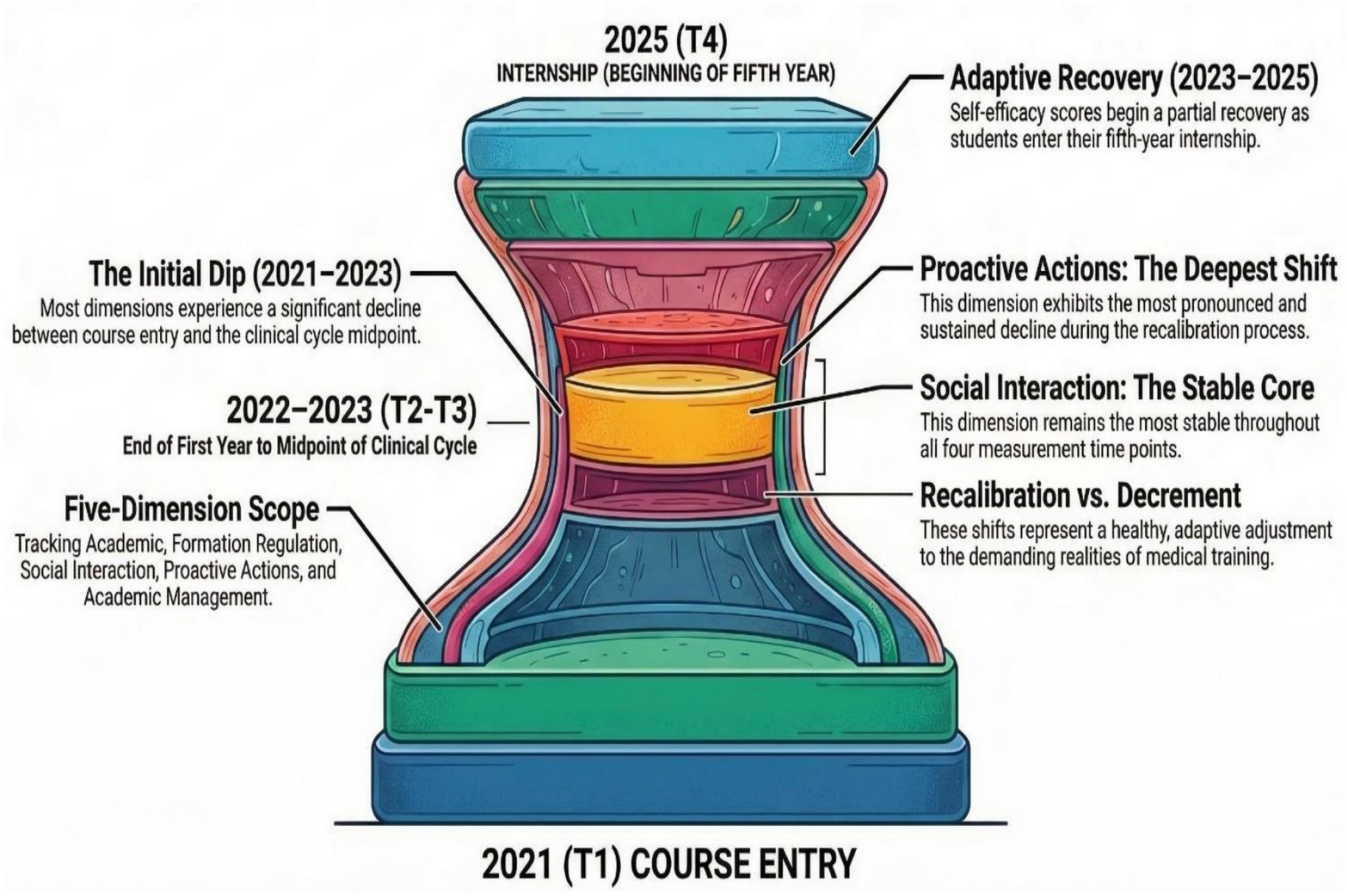
Longitudinal evolution of self‐efficacy dimensions and the adaptive recalibration process in medical students (2021–2025). Brazil, 2025. *Source:* Author.

The radar chart below (Figure 4) graphically illustrates the average trajectories of five specific self‐efficacy dimensions—academic self‐efficacy, self‐efficacy in formation regulation, self‐efficacy in social interaction, self‐efficacy in proactive actions and self‐efficacy in academic management—across key stages of undergraduate medical education in Brazil (2021, 2022, 2023 and 2025). Each distinctly coloured line represents a successive year of the study, enabling a clear visualisation of the dynamic pattern characterised by an initial significant decline, succeeded by a partial recovery. This observed trajectory is interpreted as a critical process of adaptive recalibration of self‐efficacy beliefs, essential for a more realistic self‐assessment in response to the escalating cognitive and emotional demands of medical training. Notably, the ‘self‐efficacy in proactive actions’ dimension consistently demonstrates the most substantial decline, indicative of a profound recalibration, whereas the ‘self‐efficacy in social interaction’ dimension exhibits remarkable stability throughout the longitudinal assessment.

The absence of a statistically significant association between sociodemographic variables and total self‐efficacy in longitudinal assessments (T2–T4) contrasts with the gender difference identified at baseline in self‐efficacy in social interaction, where male students exhibited higher scores with a medium effect size (*d* = 0.51). This finding is presented in Table 3. The predominance of students from private schools (74% for elementary and 76% for secondary education, as shown in Table 1) and from a specific income bracket (82% within R$ 1001.00 to R$ 3000.00) may have homogenised the sample in terms of cultural capital and access to pre‐university educational resources [24]. This relative homogeneity could explain the attenuation of initial sociodemographic differences in self‐efficacy over time, as the shared, intense experience of medical school may converge student perceptions of their capabilities within this specific demanding environment.

The observed heterogeneity of individual trajectories (Figure 3) reveals that the experience of medical undergraduate training is not uniform. While some students maintained or even increased their self‐efficacy beliefs over time, others experienced more severe and persistent shifts. This variability reinforces that individual factors such as resilience, personality, perceived social support and self‐regulated learning capacity may play a fundamental role in modulating how students navigate these developmental transitions [7]. Students with greater resilience or who employ more effective learning strategies may be better able to transform challenges into mastery opportunities [7].

If the decline in self‐efficacy is reinterpreted not as pathology but as adaptive recalibration aligned with the theoretical predictions of Dreyfus and calibration models, then educational implications fundamentally shift. The task is not to prevent or ‘correct’ the observed decline—an approach that misses its developmental necessity—but rather to systematically guide it toward healthy rather than maladaptive forms. This distinction is critical: Students must move toward conscious incompetence and realistic self‐assessment; the quality of this transition determines whether they develop robust, adaptive efficacy or disengaged, avoidant coping. Therefore, interventions must be designed as theoretical mechanisms aligned with the recalibration process itself: (1) structured mentorship providing calibrated feedback that normalises the transition from unconscious to conscious incompetence, (2) authentic mastery experiences in graduated contexts where students can build competence and consolidate new self‐beliefs, (3) peer learning environments that make the recalibration process visible and communal rather than isolating and (4) continuous monitoring to distinguish between healthy recalibration and maladaptive trajectories requiring additional support. The identification of the clinical cycle as the critical period of maximum decline (2021–2023, *d* = 1.27) and the partial recovery in internship (2025, *d* = 0.93) provides temporal landmarks for intervention placement: preventive support at entry (normalising what will come), intensive guidance during clinical transition (supporting the most acute phase) and consolidation activities during internship (reinforcing mastery and sustainable efficacy). These are not arbitrary recommendations but logically derived from theoretical predictions about the temporal signature of skill acquisition and recalibration.

*Students must move toward conscious incompetence and realistic self‐assessment*.

Ten Cate et al. [4] emphasise the development of multidimensional competencies in medical education, beyond the acquisition of technical knowledge. Interventions should aim to foster a realistic and adaptive self‐efficacy that aligns with the evolving demands of medical training [4]. Strengthening perceived environmental support, including adequate resources and institutional support, represents a crucial strategy, as environmental factors demonstrate significant association with academic satisfaction and, consequently, with student self‐efficacy [13]. Self‐efficacy contributes substantially to the preparation of medical students for clinical practice, interventions by promoting realistic self‐assessment and guiding the development of support interventions [8, 11].

Self‐efficacy assessment tools, such as the SESHE [33], could be routinely incorporated into student monitoring, enabling early identification of students who may be undergoing maladaptive recalibration or struggling to develop effective coping mechanisms during critical transitions. Bandura's Social Cognitive Theory, by focusing on the aspect of learning that occurs in a social environment, recognises and addresses the inherent complexity of the learning environment in health professions, underscoring the importance of dynamic monitoring and tailored support [5, 43].

This study presents limitations that should be considered. The retention rate of 62.5%, although comparable to similar longitudinal studies, represents significant sample attrition that may have introduced selection bias. The absence of a control group prevents definitive causal attribution of observed changes exclusively to the medical curriculum. Additionally, the exclusive use of self‐reported measures is subject to social desirability bias. Future studies should incorporate mixed methodologies, including in‐depth interviews to explore the mechanisms underlying observed changes, as well as latent trajectory analyses to identify subgroups with distinct patterns of evolution. Investigating the impact of factors such as mental health (anxiety, depression and burnout), perceived social support and objective academic performance on the trajectory of self‐efficacy would enable the construction of more complex predictive models [44, 45, 46]. Future research should also explore broader measures of outcomes beyond cognitive tests, including psychomotor skills, course retention, and career readiness, as the relationship between different dimensions of student engagement and academic performance remains controversial [14].

## 5 Conclusion

This study demonstrated a dynamic process characterised by a significant and adaptive recalibration, manifesting as a persistent decline in perceived self‐efficacy among medical students throughout undergraduate training, particularly in the capacity to engage in proactive actions, with only partial recovery during internship. The findings confirm the inherently dynamic and developmental nature of self‐efficacy and its sensitivity to the learning environment, reiterating that it is not a static characteristic but rather a construct that continuously adapts and reconfigures in response to new experiences. The magnitude of the observed decline suggests that the demands of medical undergraduate training represent a ‘reality shock’ that requires profound reassessment of individual capacities.

The results underscore that self‐efficacy decline during medical training is not an educational failure to be prevented but a theoretically predictable developmental transition requiring systematic educational design to guide toward healthy outcomes. Drawing on calibration theory and the Dreyfus model, we propose that institutional strategies must be calibrated to the theoretical mechanisms underlying recalibration: Structured psychosocial support programs should explicitly frame the expected transition from unconscious to conscious incompetence, normalising realistic self‐assessment as evidence of growth rather than deficit. Resilience development and encouragement of proactivity should target the most vulnerable dimension (self‐efficacy in proactive actions, most severely affected), providing structured opportunities for graduated mastery that rebuild efficacy within realistic developmental trajectories. Continuous formative feedback must be conceptualised not as reassurance but as calibration—helping students develop accurate self‐appraisal aligned with their actual competence. The relative stability of Social Interaction efficacy identifies peer‐based and relational interventions as particularly leverageable protective factors. Systematic incorporation of longitudinal self‐efficacy assessments enables early detection of students whose trajectories diverge from the predicted recalibration pattern, identifying those requiring intensified support to prevent maladaptive decline. In this framework, the aim is not to artificially maintain initial self‐efficacy levels or to prevent necessary decline but to facilitate healthy, adaptive recalibration that produces robust, accurate and sustained efficacy—and ultimately, confident, self‐aware competent physicians prepared for the realities of medical practice. This theoretical and evidence‐based approach transforms self‐efficacy monitoring from a surveillance tool into a developmental guidance system aligned with the predicted temporal signatures of professional skill acquisition.

## Author Contributions

**Ana Maria Rita Pedroso Vilela Torres de Carvalho Engel:** conceptualization, investigation, writing – original draft. **Weslley dos Santos Borges:** investigation. **Matheus Querino da Silva:** resources, data curation. **William Donegá Martinez:** methodology. **Samila Bernardi do Vale Lopes:** data curation. **Maysa Alahmar Bianchin:** conceptualization, resources. **Stela Regina Pedroso Vilela Torres de Carvalho:** writing – original draft, writing – review and editing. **João Daniel De Souza Menezes:** conceptualization, methodology, formal analysis, investigation. **Júlio César André:** validation, writing – review and editing, supervision, project administration, funding acquisition. **Vânia Maria Sabadoto Brienze:** resources. **Alex Bertolazzo Quitério:** conceptualization, investigation, project administration. **Emerson Roberto dos Santos:** software, validation, formal analysis, visualization. **Janaína Aparecida de Sales Floriano:** supervision, writing – review and editing.

## Funding

This research was funded by the Coordenação de Aperfeiçoamento de Pessoal de Nível Superior‐Brasil (CAPES).

## Ethics Statement

The project received approval from the FAMERP Research Ethics Committee (opinion no. 41220020.8.0000.5415), in accordance with Resolution 466/2012 of the National Health Council and the Declaration of Helsinki (World Medical Association., 2013). All participants provided written informed consent. The instruments were administered online through standardised questionnaires, with an average completion time of 15–20 min per assessment.

## Conflicts of Interest

The authors declare no conflicts of interest.

## Declaration on the Use of AI

None.

## Data Availability

The data that support the findings of this study are openly available in Open Science Framework (OSF) at https://osf.io/rpqx9/overview, reference number RPQX9.
